# Bioactivity and Bactericidal Mechanism of Histidine-Rich β-Hairpin Peptide Against Gram-Negative Bacteria

**DOI:** 10.3390/ijms20163954

**Published:** 2019-08-14

**Authors:** Na Dong, Chensi Wang, Tingting Zhang, Lei Zhang, Chenyu Xue, Xinjun Feng, Chongpeng Bi, Anshan Shan

**Affiliations:** Laboratory of Molecular Nutrition and Immunity. The Institute of Animal Nutrition, Northeast Agricultural University, Harbin 150030, China

**Keywords:** β-hairpin antimicrobial peptides, histidine, biological activity, bactericidal mechanism

## Abstract

Antibacterial peptides (APMs) are a new type of antibacterial substance. The relationship between their structure and function remains indistinct; in particular, there is a lack of a definitive and fixed template for designing new antimicrobial peptides. Previous studies have shown that porcine Protegrin-1 (PG-1) exhibits considerable antimicrobial activity and cytotoxicity. In this study, to reduce cytotoxicity and increase cell selectivity, we designed histidine-rich peptides based on the sequence template RR(XY)_2_X^D^PGX(YX)_2_RR-NH_2_, where X represents I, W, V, and F. The results showed that the peptides form more β-hairpin structures in a lipid-rich environment that mimics cell membranes. Among them, the antimicrobial peptide HV2 showed strong antibacterial activity against Gram-negative strains and almost no toxicity to normal cells. The results of our analysis of its antibacterial mechanism showed that peptide HV2 acts on the bacterial cell membrane to increase its permeability, resulting in cell membrane disruption and death. Furthermore, peptide HV2 inhibited bacterial movement in a concentration-dependent manner and had a more robust anti-inflammatory effect by inhibiting the production of TNF-α. In summary, peptide HV2 exhibits high bactericidal activity and cell selectivity, making it a promising candidate for future use as an antibiotic.

## 1. Introduction

Since the introduction of antibiotics in the 1950s, many antibiotics have been used and microbial infections can be successfully treated. Unfortunately, more and more super bacteria are developing antibiotic resistance. Therefore, we must design and identify antibacterial agents with novel mechanisms of action that can effectively overcome the mechanisms of drug resistance [1]. Antibacterial peptides (AMPs), also known as host defense peptides (HDP), are an important part of the innate immune system [2]. They have clear activities against bacteria, fungi, viruses, and parasites [3]. In addition, the antibacterial mechanism of antibacterial peptides differs from those of existing antibiotics. Studies have shown that antimicrobial peptides prevent bacterial metabolism by reorganizing the liposome domain distribution, thus causing bacterial death [4]. Antibacterial peptides destroy bacteria by physically disrupting the structure of their cell membranes, making it difficult for bacteria to easily become resistant [5,6]. Therefore, antimicrobial peptides are promising as a highly effective alternative to antibiotics. Thousands of antimicrobial peptides have been extracted from various animals and plants. However, the use of AMPs is hindered by some complex obstacles in terms of their toxicity and high manufacturing costs [7].

Previous studies have found that amphiphilic β-hairpin antibacterial peptides have high antibacterial potential and cell selectivity [8,9]. Protegrin-1 (PG-1) has a typical β-hairpin structure and has strong antibacterial activity against Gram-positive and Gram-negative bacteria [10]. However, PG-1 also has high cytotoxicity and hemolytic activity, which hinders its development as a candidate antimicrobial agent [11]. However, studies have shown that PG-1 might serve as a useful nontoxic stable scaffold for future therapeutic peptide analogs [12]. Previous work has shown that replacing lysine residues with histidine residues can reduce the cytotoxicity of histidine-containing peptides and that most of the peptides maintain excellent antibacterial activity [13]. Moreover, histidine-containing peptides have the advantages of good stability and high cell selectivity [14]. Therefore, histidine-containing AMPs are good candidates for use in treatment. Based on the study of the structure and function of PG-1 and symmetrical terminal peptides, new designs define “PG” as a β-turn, and the hydrophobic amino acids and the positively charged amino acids are alternately arranged to obtain symmetry in the β-hairpin structure. The sequence of the designed peptides is RR(XY)_n_X^D^PGX(YX)_n_RR-NH_2_, (*n* = 2, X represents I, W, V, and F). I, W, V, and F are hydrophobic amino acids; more specifically, F and W are hydrophobic aromatic and heterocyclic amino acids, respectively, while I and V are aliphatic amino acids. Y is a positively charged H amino acid residue. When *n* = 2, symmetrical-end polypeptides show higher antimicrobial efficacy compared with that of PG-1 [7]. Furthermore, since arginine residues are electrostatically attracted to the bacterial cell membrane and can bind the peptides to the phospholipid membrane to enhance their antibacterial activity [15,16], two arginine residues are added to the symmetric ends of the peptides. Finally, the amidation of the C-terminus increases the positive charge of the peptides and increases their structural stability, thus facilitating their translocation into the bacterial inner membrane. This interaction enhances the antimicrobial activity of the peptides and improves their stability [17,18]. The purpose of this study was to develop and synthesize peptides to find effective AMPs with strong antibacterial activity and selectivity.

## 2. Results

### 2.1. Design and Characterization of the Peptides

Previous studies showed that amphiphilic β-hairpin antibacterial peptides have high antibacterial potential and cell selectivity [9,19]. Replacement of lysines with histidines results in histidine-containing peptides with reduced cytotoxicity, and most of these peptides maintain excellent antibacterial activity [13]. Furthermore, the tight binding of arginine to the phospholipid membrane helps to increase the antibacterial activity of the β-hairpin antimicrobial peptide PG-1 [20]. Therefore, we designed a series of histidine-rich β-hairpin-like antimicrobial peptides according to the sequence template RR(XH)_2_X^D^PGX(HX)_2_RR-NH_2_, where X represents I, W, V, and F (Table 1).

The molecular weights of all of the peptides were consistent with their theoretical values, indicating that the peptides were successfully synthesized (Supplementary Figure S1–S2). The hydrophobicities of the peptides in solution were reliably mirrored by their high-performance liquid chromatography (HPLC)retention times. The retention times for peptide HI2, HW2, HV2, HF2, and PG-1 were 11.13, 10.55, 12.20, 18.06 and 12.56 min, respectively, indicating the following hydrophobic order: HF2 > PG-1 > HV2 > HI2 > HW2(Table 1).

The relative hydrophobic moments (Table 1) indicate that HI2 (2.39), HW2 (2.35), HV2 (1.89), and HF2 (2.53) have a better balance between the hydrophobic phase and the hydrophilic phase compared with that of PG-1 (1.37).

### 2.2. CD Analysis

**Figure 1:**
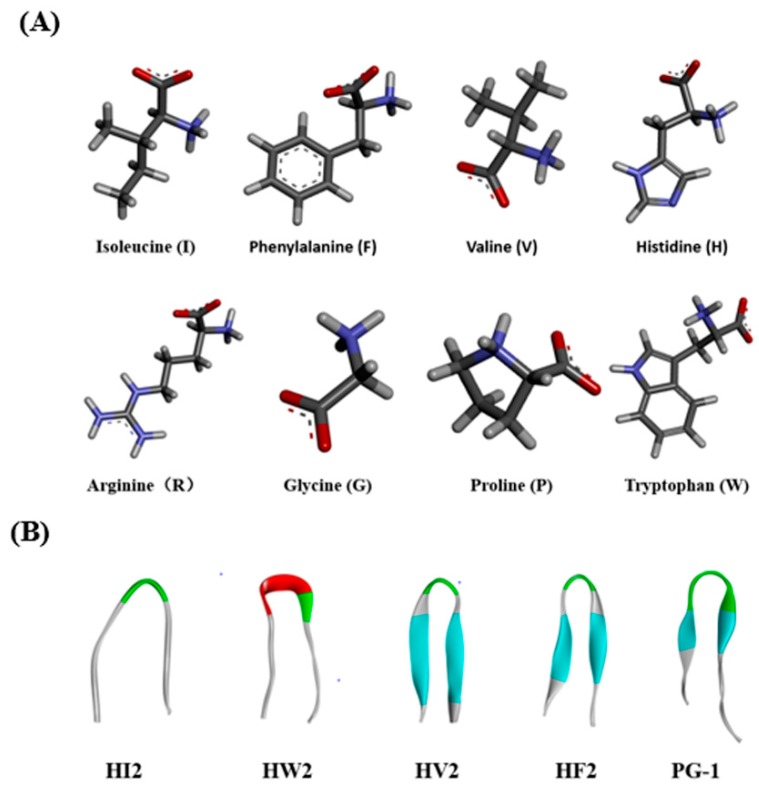
(**A**) Molecular three-dimensional structure of I, F, V, H, R, G, P, and W. (**B**) Peptides three-dimensional structure projections of HI2, HW2, HV2, HF2, and PG-1.

**Figure 2:**
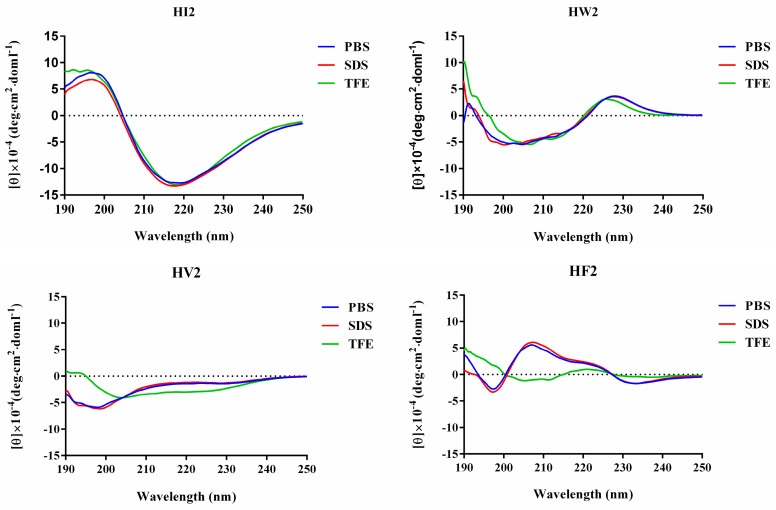
The CD spectra of the peptides. The peptides were dissolved in 10 mM sodium phosphate buffer (pH 7.4) (Blue line), 30 mM SDS (Red line) and 50% TFE (Green line). The mean residue ellipticity was plotted against wavelength. The values from three scans were averaged per sample, and the peptides concentrations were fixed at 150 μM.

The three-dimensional structures were predicted using I-TASSER, and the designed peptides showed similar β-hairpin structures (Figure 1B). All four peptides were subjected to circular dichroism (CD) spectroscopy in PBS (pH 7.4), 30 mM SDS (mimicking the microbial membrane environment) and 50% TFE (mimicking the hydrophobic environment) (Figure 2). The spectrum of peptide HI2 showed significantly more β-sheets in the three solutions, showing a positive peak at 195–200 nm and a negative peak at 220 nm [21]. In the sodium phosphate buffer and SDS, HW2, HV2 and HF2 exhibited free-curled secondary structures. In TFE, HW2, HV2 and HF2 showed a conformational transition, showing a tendency to adopt β-hairpin conformations, characterized by a negative ellipticity of approximately 205 nm and an intersection at 200 nm [22].

### 2.3. Hemolytic Activity

**Figure 3:**
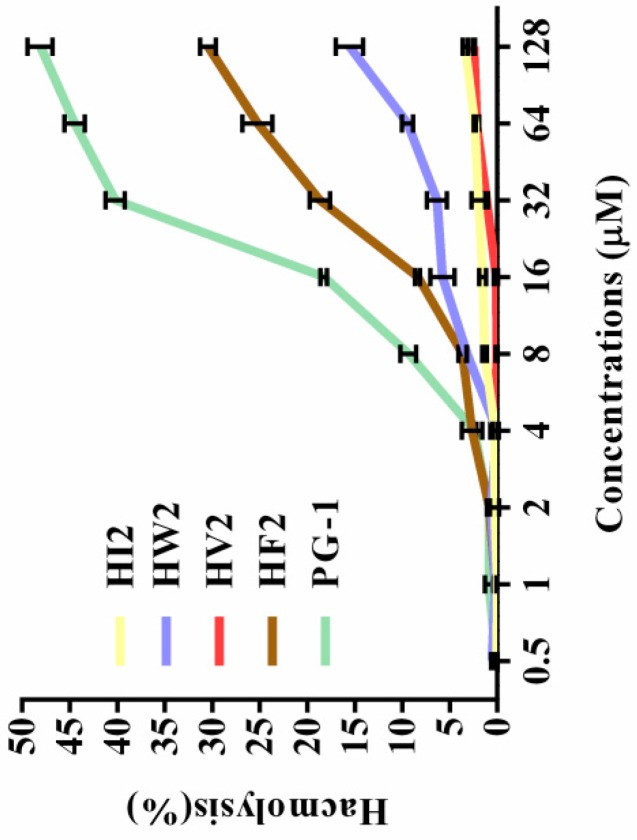
Hemolytic activity of peptides against hRBCs. The peptides were incubated with hRBCs in a 96-well microtiter plate and measured for optical density (OD) at 570 nm. For negative and positive controls, hRBCs in PBS and 0.1% Triton X-100 were used, respectively.

The toxicities of the peptides to eukaryotic cells were assessed by measuring their ability to lyse human erythrocytes at peptide concentrations ranging from 1 to 128 μM. Table 2 gives the minimum hemolytic concentration of each peptide, the concentration that causes 5% lysis of human erythrocytes. The hemolytic activities of the peptides at various concentrations are plotted in Figure 3. The hemolysis rates of the five peptides (HI2, HW2, HV2, HF2 and PG-1) were 3.4%, 15.58%, 2.48%, 30.46%, and 48.13%, respectively, at the highest concentration (128 μM). These results indicate that peptide HV2 had a lower hemolytic activity in the presence of bactericidal activity. The PG-1 peptide was highly hemolytic compared to the novel peptides.

### 2.4. Cytotoxicity

**Figure 4:**
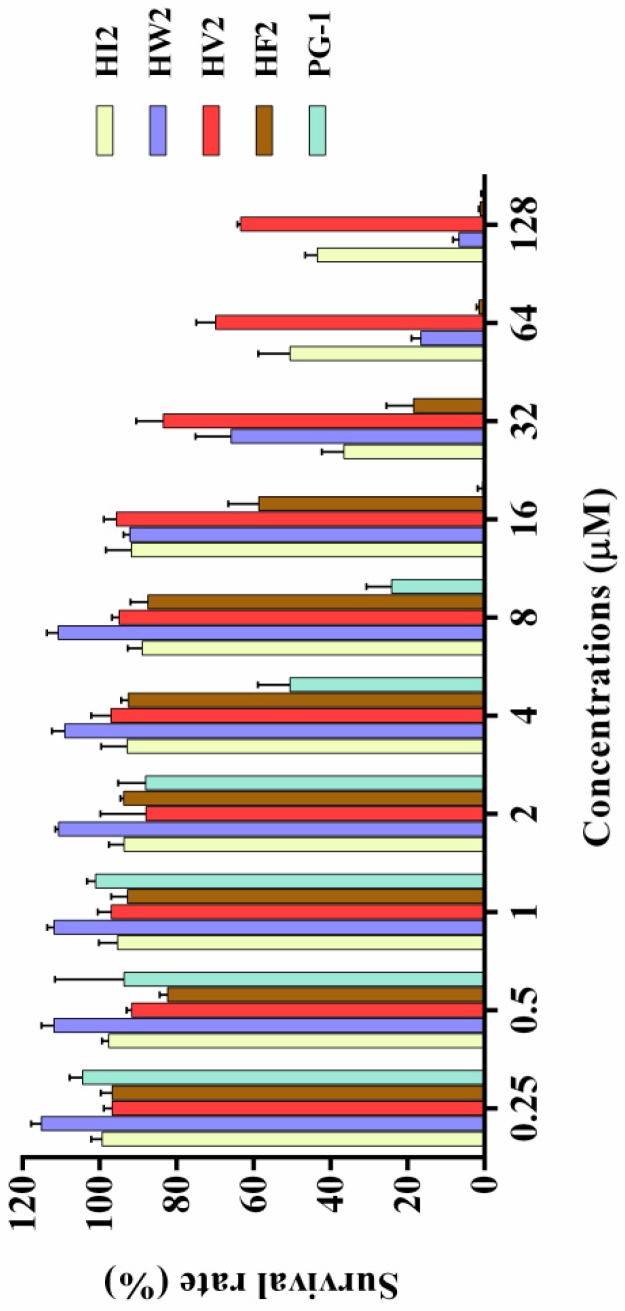
Cytotoxicity of peptides against RAW264.7. The peptides were incubated with RAW264.7 in a 96-well microtiter plate, and after incubation with MTT, the supernatant was discarded and dimethyl sulfoxide was added and the optical density (OD) at 570 nm was measured.

The effects of the antimicrobial peptides on the survival rate of RAW264.7 murine macrophages and HEK 293T cells were determined via cell culture assays to evaluate their toxicities against normal mammalian cells. The graph in Figure 4 shows the effects of the newly designed β-hairpin antimicrobial peptides on the survival rate of mammalian cells based on the cell culture assay. With increasing concentrations, the β-hairpin antibacterial peptides decreased the survival rates of the mouse monocyte macrophages to varying degrees. HV2 slowly reduced the survival rate of the mouse monocyte macrophages, and the survival rate was 66% at the highest concentration (128 μM). These β-hairpin antibacterial peptides were not very toxic to mammalian cells. As the concentrations of HW2, HF2, and PG-1 increased, the survival rates of normal mammalian cells significantly decreased, and the cytotoxicity increased. When the concentrations of HW2, HF2, and PG-1 reached 128 μM, the survival rates of the mammalian cells were reduced to 6%, 1% and 1%, respectively, indicating that these peptides had strong toxic effects on mammalian cells. In addition, we tested the toxicity of HEK 293T cells and found that when HV2 and PG-1 concentrations were 128 μM, the survival rates of cells was 75% and 21%, respectively (Supplementary Figure S3). These results indicate that HV2 is less cytotoxic to mammals.

### 2.5. Antimicrobial Activity

The antimicrobial activities of various peptide concentrations against Gram-negative and Gram-positive strains are summarized in Table 2. HI2 was inactive against Gram-positive bacteria and had lower activity against Gram-negative bacteria. HW2 and HF2 showed higher activity against Gram-positive and Gram-negative bacteria. HV2 showed antimicrobial activity against Gram-negative bacteria, with an MIC of 4–8 μM, but showed no antibacterial activity against Gram-positive bacteria at the tested concentrations. To further assess the cell selectivity of the peptides, the therapeutic index (TI), defined as the ratio of the MHC to the geometric mean (GM), of each peptide was calculated. As shown in Table 2, we found that HV2 had the highest TI among the tested peptides. Against Gram-negative bacteria, the TI of HV2 was as high as 35.9, which was 28 times that of PG-1. These results indicate that HV2 was more selective for bacterial cells than for human erythrocytes. Therefore, HV2 has greater application potential.

### 2.6. Salt Sensitivity

To evaluate the in vivo stability of the antibacterial peptides, we chose to treat *E. coli* cells with designed peptides in the presence of different physiological concentrations of salt ions. The tolerance of the antimicrobial peptides to salt ions was determined by measuring the magnitude of the antibacterial activity of the antimicrobial peptides after treatment. Through these experiments, we found that salt ion concentrations closest to those under physiological conditions had little effect on the activities of the antimicrobial peptides and could even have a promoting effect. Also, 8 μM Zn^2+^ increased the efficacy against Gram-negative bacteria [23]. These results showed that HV2 tolerates physiological salt concentrations effectively.

### 2.7. Antibacterial Mechanism Study

#### 2.7.1. LPS Binding Assay

**Figure 5:**
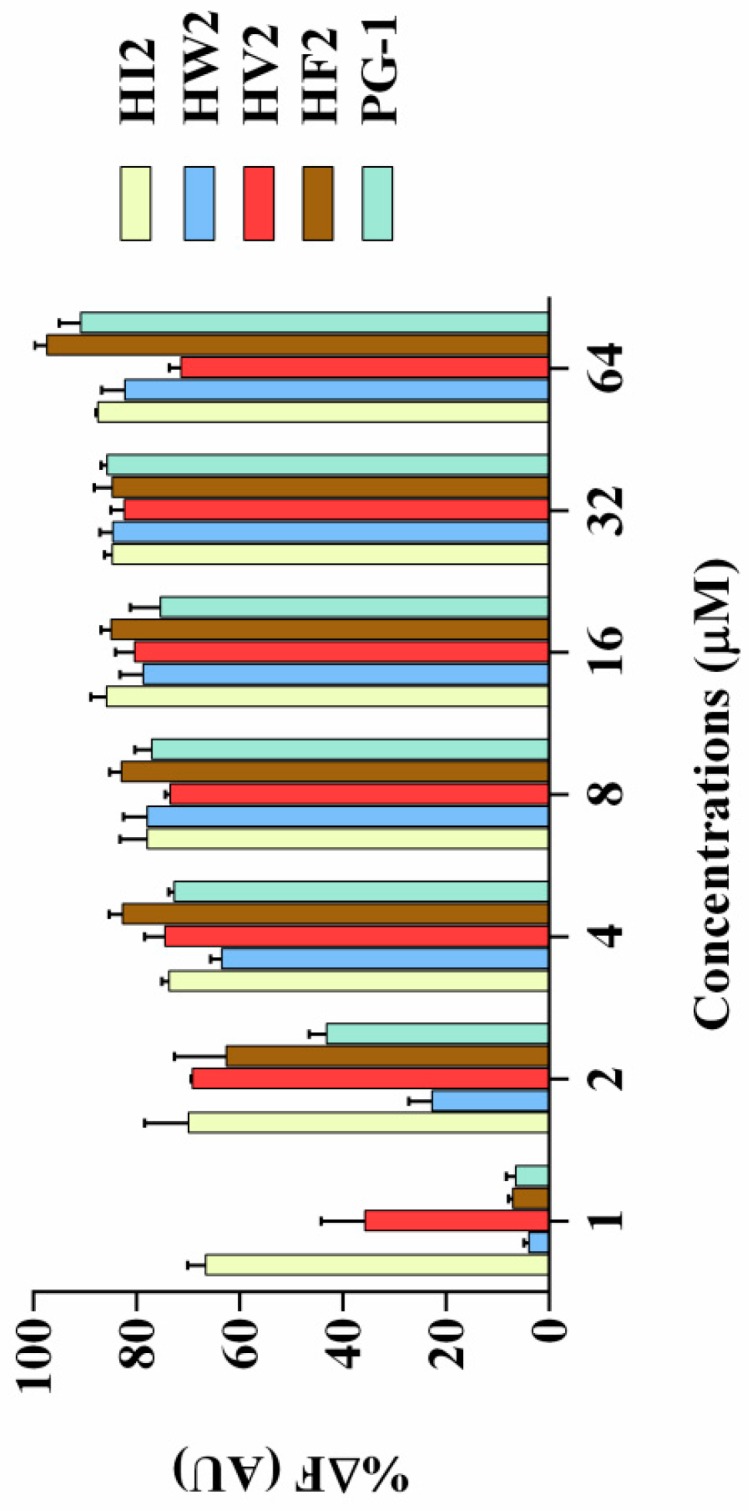
The combining ability of HI2, HW2, HV2, HF2, and PG-1 (1 μM, 2 μM, 4 μM, 8 μM, 16 μM, 32 μM, and 64 μM) with LPS. 50 μg/mL LPS from E. coli O111:B4 was incubated with 5 μg/mL BODIPY-TR cadaverine in Tris buffer (50 mM, pH 7.4) for 4 h at room temperature. Subsequently, the peptides were serially diluted in Tris buffer and incubated with equal volumes of the LPS-probe mixture for 1 h in a 96-well black plate. The fluorescence was measured (excitation λ = 580 nm, emission λ = 620 nm) on a spectrofluorophotometer.

Lipopolysaccharide (LPS) is the main component of the Gram-negative bacterial cell wall. When abundant lipopolysaccharide is present in the blood, it can cause sepsis and septic shock [24,25]. As shown in Figure 5, compared with that of PG-1, the four peptides (HI2, HW2, HF2 and HV2) had higher LPS binding ability. When the concentration of these four peptides was higher than 4 μM, the LPS binding ability of the peptides was higher than 60% of the binding activity of polymyxin B.

#### 2.7.2. Outer Membrane Permeability Assay

**Figure 6:**
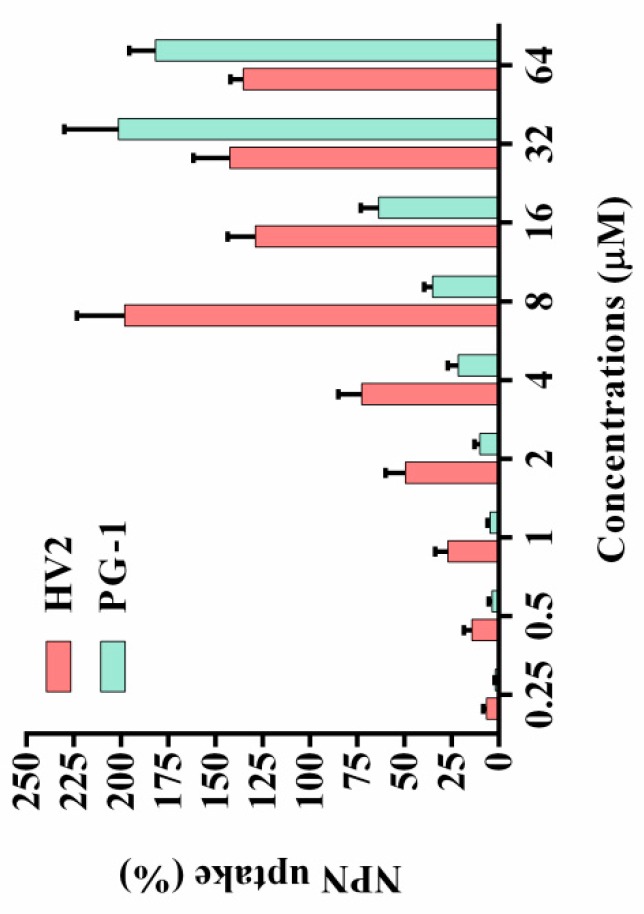
Outer membrane permeabilization assays of HV2 and PG-1. The uptake capacity of different concentrations of peptides to the NPN of *E. coli* ATCC 25922 was evaluated using a fluorescent dye (NPN) assay. The NPN uptake was monitored at an excitation wavelength of 350 nm and an emission wavelength of 420 nm. Polymyxin B was used as a positive control for its strong outer membrane permeability properties.

Most natural antimicrobial peptides primarily act on the bacterial cell membrane [26]. The outer cell membrane provides an additional layer of protection for the organism without affecting the exchange of substances required for life. At physiological pH, the outer cell membrane has a negative net charge, which increases the ability of cationic antimicrobial peptides to bind to the outer cell membrane [27,28]. Therefore, the ability of the peptides to permeabilize the outer membrane was determined via the N-phenyl-1-naphthylamine (NPN) uptake test. The hydrophobic fluorophore NPN is normally excluded by the outer membrane; however, NPN is absorbed upon outer membrane permeabilization, resulting in cells with increased fluorescence intensity. As shown in Figure 6, peptide concentrations from 0.25 to 64 μM were required to permeabilize the outer membrane of *E. coli* ATCC25922. The only exception was that the outer membrane permeabilization of 8 μM HV2 was stronger than that at its high concentration, which may be due to its higher hydrophobicity. At high concentrations, aggregation and sedimentation were more likely to occur, thereby affecting the ability the ability of the peptides to penetrate the outer membrane. In the present study, the outer membrane permeabilization by HV2 was stronger than that by PG-1.

#### 2.7.3. Inner Membrane Permeability

**Figure 7:**
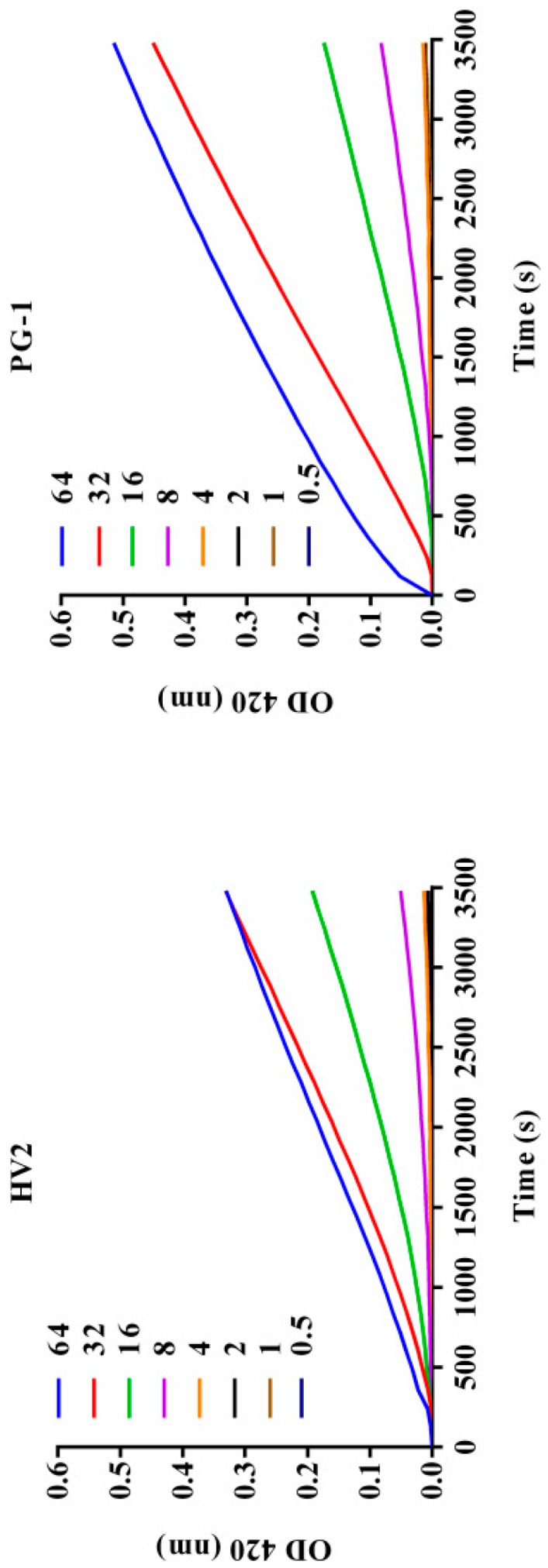
Inner membrane permeability of the peptides. Hydrolysis of ONPG due to release of cytoplasmic β-galactosidase of *E. coli* ATCC25922 treated with peptides HV2, and PG-1 at a series of different concentration (0.5 μM, 1μM, 2 μM, 4 μM, 8 μM, 16 μM, 32 μM, and 64 μM) was measured spectroscopically at an absorbance of 420 nm as a function of time. The control was measured without the peptides.

The interaction between the antimicrobial peptide and the bacterial cell membrane was further evaluated by measuring the β-galactosidase activity of the bacterial cells. When the *E. coli* cell membrane is destroyed by an antibacterial substance, β-galactosidase is released from the cells where it can degrade ONPG to produce o-nitrophenol, which has an absorbance at 420 nm. As shown in Figure 7, HV2 and PG-1 triggered rapidly increase permeability from 0 s to 3480 s. The results of this experiment indicate that the inner membrane permeabilization by HV2 is similar to that of PG-1.

#### 2.7.4. Membrane Depolarization

**Figure 8:**
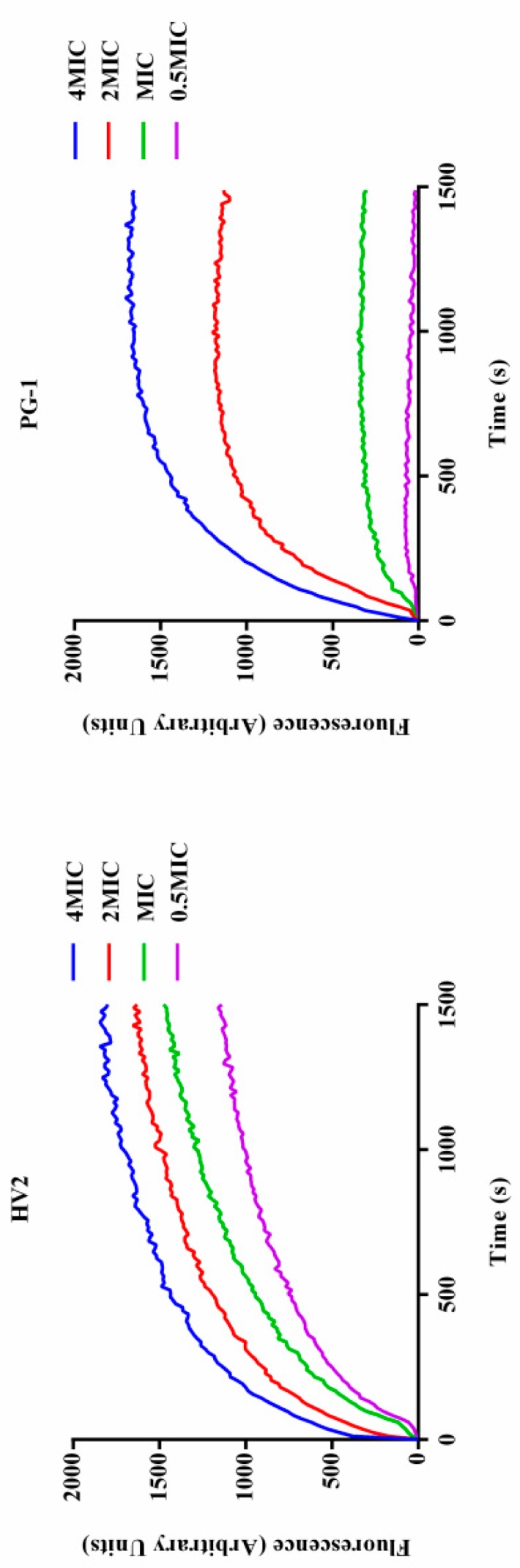
Cytoplasmic membrane depolarization of *E. coli* ATCC25922 was measured using the membrane potential-sensitive dye diSC_3_-5.

When the bacterial inner membrane is disturbed by antimicrobial peptides, ion channels are generated, and, after membrane depolarization, diSC_3_-5 dye accumulated in the bacterial membrane in its quenched state can enter the solution such that its fluorescence intensity increases. As shown in Figure 8, these two peptides induce sustained diSC_3_-5 release over 1500 s, and over 1500 s, each peptide showed dose-dependent effects. In summary, HV2 causes more rapid and stronger plasma membrane depolarization relative to PG-1.

#### 2.7.5. Swimming Motility Analysis

**Figure 9:**
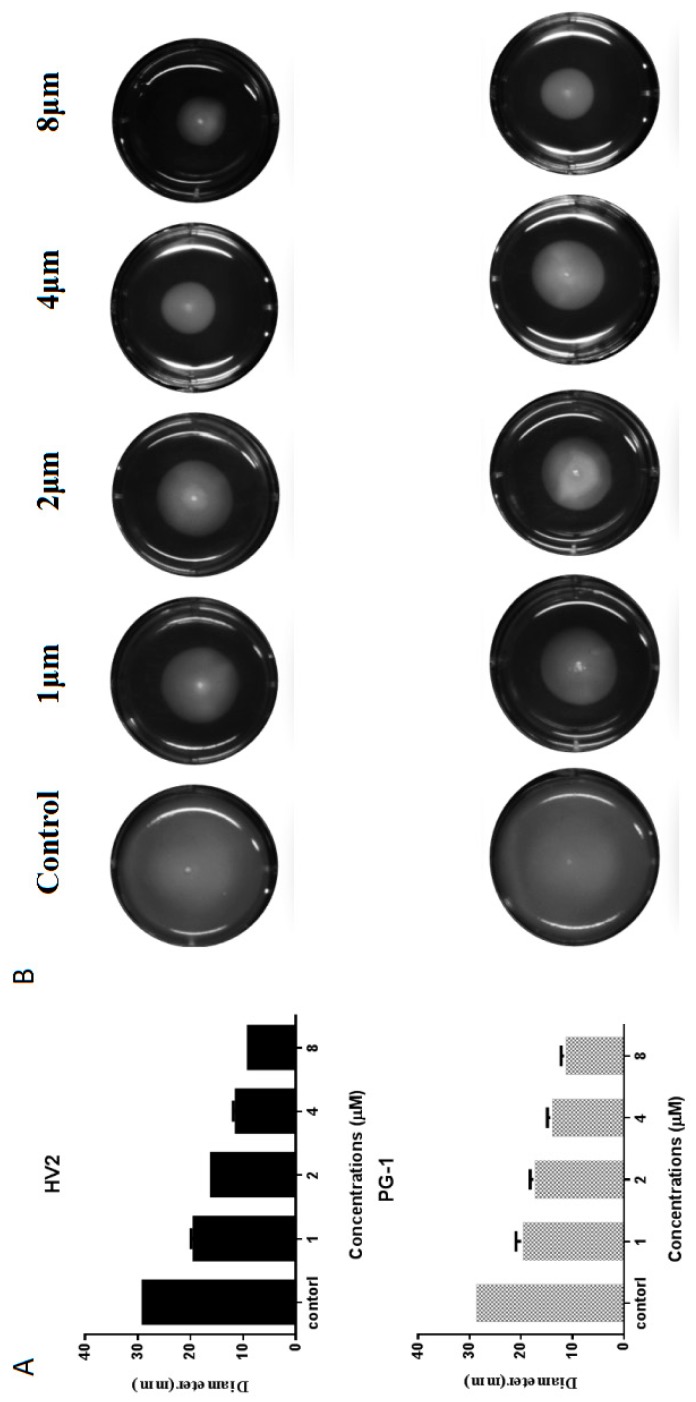
Swimming motility of *E. coli* ATCC25922 treated with HV2 and PG-1. Swim plates were prepared using 0.3% agar and inoculated from overnight cultures standardized to an optical density at 600 nm of 1.0. Images were taken after 20 h incubation at 37 °C. (**A**) the diameter of the swimming bacteria circle; (**B**) swimming bacteria circle.

Swimming motility is a flagella-dependent bacterial movement that is controlled by the respiratory chain on the plasma membrane. When the plasma membrane potential or proton motive force (PMF) are disrupted, the entire respiratory electron transport chain is restrained, resulting in decreased ATP synthesis, which is very important for flagella function [28]. Therefore, we studied the effects of HV2 and PG-1 on the swimming movement of *E. coli* ATCC25922 by monitoring the bacterial movement on low viscosity swimming plates (order 0.3%, *b*/*v*). Surprisingly, HV2 and PG-1 significantly reduced the diameter of the swimming bacteria relative to that of the untreated control (Figure 9B). Furthermore, both peptides suppressed bacterial swimming in a dose-dependent manner (Figure 9A).

#### 2.7.6. SEM

**Figure 10:**
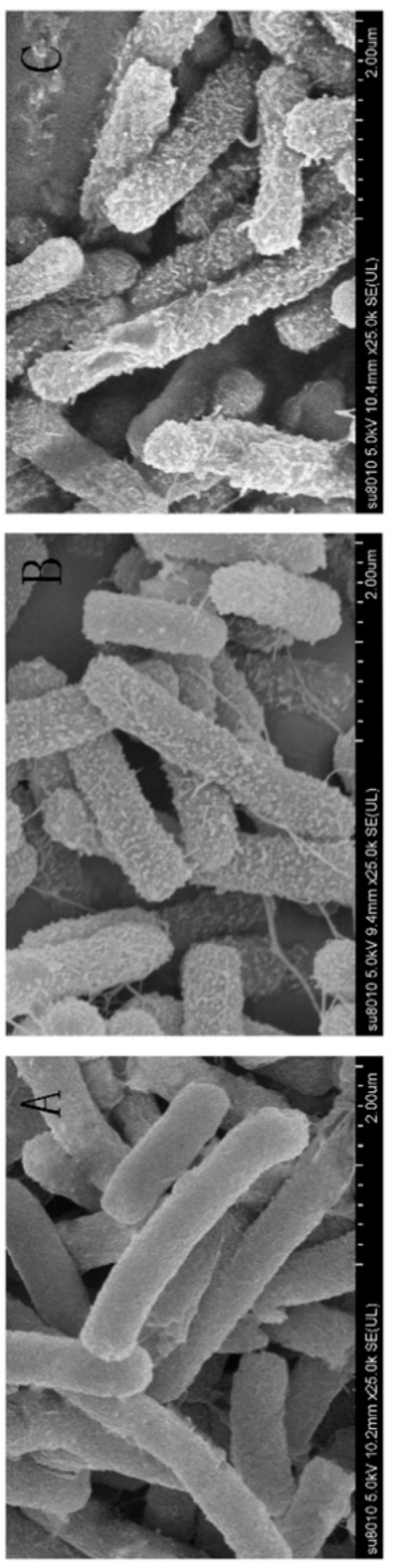
Scanning electron micrographs of E. coli ATCC25922 treated with HV2 and PG-1. (**A**) Control, no peptides; (**B**) HV2-treated, 8 μM; (**C**) PG-1-treated, 4 μM. Bacteria were treated with peptides at 1 × MIC for 1 h.

After HV2 treatment, we could directly observe cell morphology changes via SEM (Figure 10). Figure 10B and Figure 10C show SEM images of *E. coli* ATCC25922 after treatment with HV2 and PG-1 for 1 h at 1 × MIC. Control cells not treated with peptides showed a smooth and bright surface (Figure 10A), while the membrane surface of the *E. coli* cells became rough and wrinkled after treatment with 1 × MIC HV2 and PG-1.

#### 2.7.7. Inhibition of Endotoxin-Induced Inflammation

**Figure 11:**
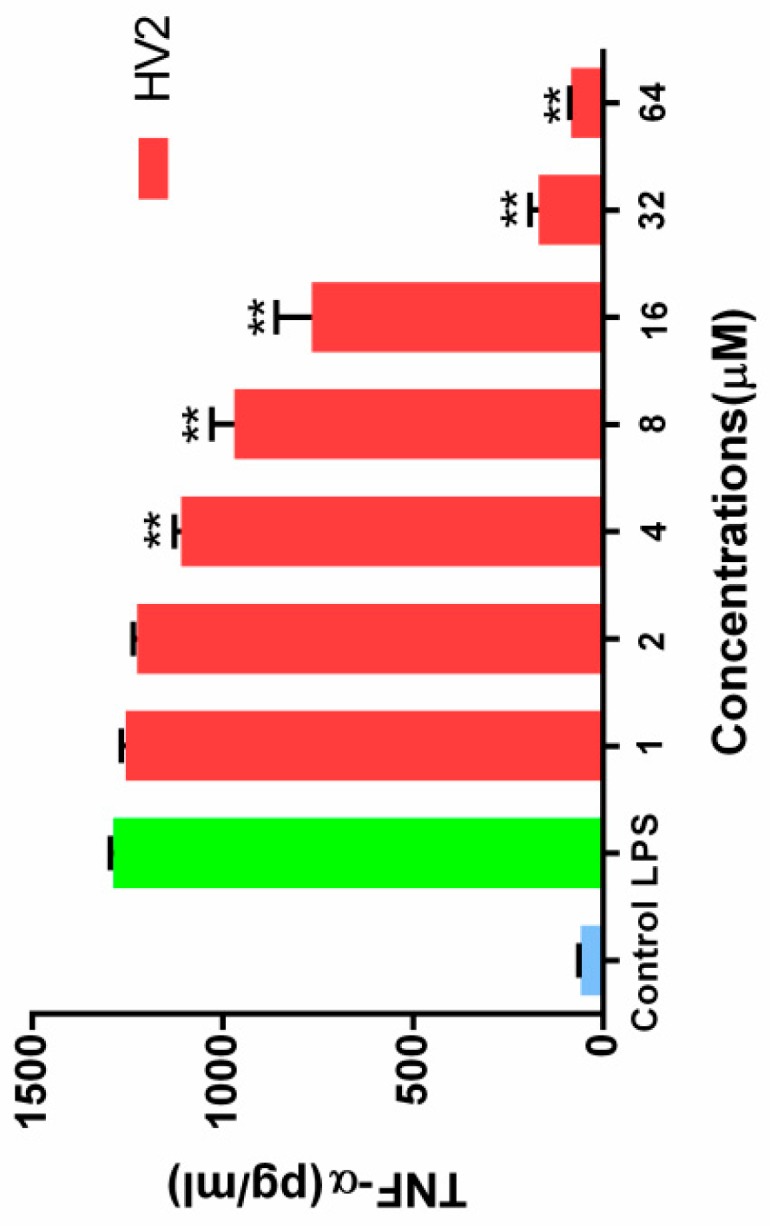
Effects of HV2 on LPS-stimulated TNF-α production in RAW 264.7 cells. Amounts of TNF-α in cell culture supernatants were determined by ELISA and the Griess reagent, respectively. Data shown are the mean ± SEM of three independent experiments: (∗∗) *p* < 0.01, compared to the LPS-alone treated.

When RAW264.7 cells were stimulated with 100 ng/mL LPS, the endotoxin neutralization ability of HV2 was further examined in the absence or presence of HV2 at different concentrations. The neutralizing activity of HV2 against LPS was determined by measuring the level of tumor necrosis factor TNF-α, which is a typical inflammatory mediator. At a peptide concentration of 32 μM, HV2 had almost 100% neutralizing activity against LPS. The results indicate that HV2 has very significant LPS neutralizing activity (Figure 11).

## 3. Discussion

Antimicrobial peptides (AMP), which act primarily via membrane altering mechanisms, have become highly anticipated antimicrobial agents with great potential to overcome the problem of antibiotic-induced resistance. However, some natural antimicrobial peptides can destroy mammalian cell membranes, resulting in hemolysis and cytotoxicity and, thus, a reduction in the cell selectivity of the peptides [29]. To overcome these inherent disadvantages, techniques have been employed to optimize peptide sequences to increase their biological activity, particularly their cell selectivity and in vivo stability. Template-assisted methods are potential strategies for the development of synthetic antimicrobial peptides that differ from natural immunity-associated antimicrobial peptides that might damage the body in large-scale clinical applications. Therefore, we designed a series of histidine-rich β-hairpin-like antimicrobial peptides according to the sequence template RR(XH)_2_X^D^PGX(HX)_2_RR-NH_2_, where X represents the hydrophobic amino acids I, W, V, F (Table 1).

In the present study, we used the amino acid G, the hydrophilic amino acids R, and H, as well as five hydrophobic amino acids I, F, V, P, and W, which exhibited different three-dimensional structures (Figure 1A). Peptides with β-chains formed with the aid of different amino acids had varying levels of amphiphilicity, and the peptide HV2 had a more robust antibacterial capability compared with that of peptide PG-1 amphiphilic systems (Table 1). In this study, we used CD spectroscopy to study the most common conformations of the peptides in a water environment (PBS) and in membranous environments (SDS, TFE) (Figure 2). The results indicate that the antimicrobial peptides of different types of amino acids have significantly different secondary structures. Since the aliphatic amino acid V is rather flexible, it caused an increase in the flexibility of HV2 in a hydrophobic environment and led to a β-hairpin conformation. A predictive software program predicted that HV2 might show the hairpin and sheet conformation consistent with its structure in TFE. (Figure 1B).

Antimicrobial assays showed that HF2, HV2 and HW2 had strong antibacterial activity. Within a certain range, the antibacterial activity of antimicrobial peptides is determined by their hydrophobicity, which may additionally explain why highly hydrophobic HF2 had a strong antibacterial effect. The hydrophobicities of peptides are commonly measured by measuring the retention time of the peptides in a reversed-segment substrate [30]. The most amphiphilic peptide, HF2, also exhibited high antimicrobial activity. However, HV2 had strong antibacterial activity against Gram-negative bacteria and almost no bactericidal activity against Gram-positive bacteria. It has been shown that the double membrane structure of Gram-negative bacteria is a weaker barrier compared to the mono-membrane and thick peptidoglycan layers of Gram-positive bacteria; therefore, Gram-negative bacteria are more susceptible than Gram-positive bacteria [31]. However, the antimicrobial activity of antimicrobial peptides is not the only criterion for evaluating their potential therapeutic value. The main factor limiting the therapeutic value of antimicrobial peptides is their toxicity to mammalian cells. In respect to their hemolytic activity (Figure 3, Table 2), even at the highest concentration of 128 μM, the hemolysis rate of HV2 was less than 5%. However, HW2 and HF2, which have higher activities compared with that of HV2, had hemolysis rates of 15.58% and 30.46%, respectively, at the highest concentration (128 μM). As described above, HV2 showed stronger specific selectivity against Gram-negative bacteria without blood activity. HV2 showed an optimal balance between its antimicrobial and hemolytic activities. As shown in Figure 4, HV2 was not cytotoxic at concentrations below 128 μM, reflecting a better selectivity for negatively charged phospholipid membranes versus the zwitterionic phospholipids of mammalian cell membranes.

Next, the effects of physiological salt concentrations on the antimicrobial activities of these peptides were investigated. Interestingly, in salt ion solution, the bactericidal activities of the antimicrobial peptides were not reduced and even increased in some cases (Table 3). Many papers have demonstrated that the charge screening effect of Na^+^ hinders electrostatic interactions between the peptides and the bacterial membrane [5,32], thus leading to decreased antimicrobial activity. Some studies have shown that divalent or multivalent cations not only reduce the activity of AMPs by interfering with electrostatic interactions but also by competing against peptides and cations at membranes that interact with LPS [33]. Thus, HV2, with its higher net positive charge, had a higher affinity to the bacterial membrane and overcame the influence of divalent cations at low physical cation concentrations.

Antimicrobial peptides may disrupt cell membrane integrity or cross the cell membrane into the cytoplasm to interact with intracellular targets to exert their antimicrobial effects [18,34]. Positively charged AMPs accumulate on the surface of the anionic bacterial membrane via electrostatic interactions. Above this critical concentration, the hydrophobic portion of the AMP is directly inserted into the membrane, thus leading to membrane depolarization, membrane lysis and cell death [35]. In this study, HV2 and the LPS on the outer membrane surface of the Gram-negative bacterial cells can efficiently bind to each other (Figure 5), thereby promoting the binding of the antimicrobial peptides to the membrane. This effect also explains why the biological activity of HV2 against Gram-negative bacteria was higher than that against Gram-positive bacteria. HV2 showed highly efficient outer membrane permeabilization similar to that of PG-1 (Figure 6), and it showed higher intimal permeability at different concentrations (Figure 8). Permeabilization and perturbation of the microbial membrane via pore formation and ion channel generation, which lead to potential changes, ultimately results in dissipation of the plasma membrane [36]. Positively charged AMPs inhibit ATP synthesis and flagellar movement, thereby reducing or preventing the swimming activity of *E. coli* ATCC25922 (Figure 9) [37]. Our SEM studies further demonstrated that HV2 causes significant vesicles on the outer membrane (Figure 10), indicating that HV2 causes robust damage to the cell envelope. Thus, HV2 exerts its bactericidal activity by forming pores in the membrane that lead to leakage of the cytosol and eventually to cell lysis.

Gram-negative bacteria and lipopolysaccharide (LPS) can induce sepsis. Furthermore, they can cause infectious diseases that pose major threats to humans and animals [38]. Lipopolysaccharide (LPS; endotoxin) is a strong immunostimulant that is released on the Gram-negative outer membrane [39,40]. LPS stimulates immune cells to release inflammatory cytokines such as TNF-α [41]. An excess of inflammatory cells can cause shock or even death [42]. Previous studies have shown that AMP has a strong endotoxin neutralizing effect via its ability to directly bind to LPS or to block the binding of LPS to LPS binding protein (LBP) [43,44]. HV2 also inhibited the production of TNF-α by LPS in a concentration-dependent manner (Figure 11). These results indicate that HV2 probably inhibits the production of cytokines by these cells via the effective binding activity of LPS, thereby blocking the binding of LPS to RAW.246.7 cells to protect against endotoxin shock [45].

The results of the above experiments showed that HV2 could inhibit the activity of Gram-negative bacteria not only by binding and destroying their membrane [14] but also by inhibiting flagellar movement [46]. HV2 could also inhibit the production of cellular inflammatory factors induced in response to the Gram-negative bacteria membrane. Therefore, HV2 has great potential for use in treating infectious diseases caused by Gram-negative bacteria.

## 4. Materials and Methods

### 4.1. Materials

The bacterial strains *E. coli* K88, *E. coli* K99, *E. coli* ATCC25922, *S. epidermidis* ATCC12228, *P. aeruginosa* ATCC27853, *S. aureus* ATCC29213, *S. pullorum* C7913, and methicillin-resistant *S. aureus* ATCC43300, were provided by the College of Veterinary Medicine, Northeast Agricultural University (Harbin, China). *E. coli* UB1005 was presented by the State Key Laboratory of Microbial Technology, Shandong University (Jinan, China). The human red blood cells (hRBCs) were provided by healthy donors. The murine RAW264.7 macrophage cell line and HEK 293T cells were provided by the College of Animal Science and Technology, Northeast Agricultural University (Harbin, China).

Potassium chloride, sodium chloride, zinc chloride, ferric chloride, magnesium chloride, ammonium chloride, and phosphate-buffered saline (PBS) were all purchased from Kermel (Tianjin, China). Bovine serum albumin (BSA), trifluoroethyl alcohol (TFE), Triton X-100, O-nitrophenyl-b-d-galactopyranoside (ONPG), lipopolysaccharide (LPS, derived from *E. coli* O55:B5), dimethyl sulfoxide (DMSO), polymyxin B, 3-(4,5-Dimethyl-2-thiazolyl)-2,5-diphenyltetrazolium bromide (MTT), N-phenyl-1-naphthylamine (NPN), 3,3, dipropylthiadicarbocyanine (diSC_3_-5), 4-(2-hydroxyethyl)piperazine-1-ethanesulfonic acid (HEPES), acetone (analytical grade, 99%), glutaraldehyde (synthetic grade, 50% in H_2_O), tertiary butanol (analytical grade, 99%), and ethanol (analytical grade, 99%) were all purchased from Sigma-Aldrich (Shanghai, China). Sodium dodecyl sulfate (SDS) was obtained from Amresco (Wayne, PA, USA). Dulbecco’s modified Eagle’s medium-high glucose (DMEM) and fetal bovine serum (FBS) were obtained from Invitrogen Corporation (Carlsbad, CA, USA). Lactose and glucose (analytical grade) were ordered from Zhiyuan (Tianjin, China). Mueller Hinton Broth (MHB) powder, yeast extract, tryptone, and agar were purchased from AoBoX (Shanghai, China).

### 4.2. Peptide Synthesis and Sequence Analysis

The designed peptides were synthesized and purified by GL Biochem (Shanghai, China). Acetonitrile was used on an analytical Kromasil C18 column (4.6 mm × 250 mm, 5.0 μm, LC 3000, Beijing, China), and the purity of the peptides was determined to be greater than 95% via reversed-phase high-performance liquid chromatography (RP-HPLC) with a flow rate of 1.0 mL/min. The experimental molecular weights of the peptides were evaluated via ESI-MS analysis, and these values were very close to the calculated masses. Next, the peptides were dissolved in deionized water at a concentration of 2.56 mM, and the peptide solutions were stored at −20 °C.

We calculated the main physical and chemical parameters and sequences of engineered peptides via the bioinformatics program ProtParam (http://www.expasy.org/tools/protparam.html). The mean hydrophobicities and relative hydrophobic moments were calculated online using CCS scale (http://www.bbcm.univ.trieste.it/~tossi/HydroCalc/HydroMCalc.html). The three-dimensional structure predictions were predicted using I-TASSER (http://zhanglab.ccmb.med.umich.edu/I-TASSER/).

### 4.3. Circular Dichroism (CD) Measurements

The peptides were dissolved in 10 mM PBS, 30 mM SDS and 50% TFE to a final concentration of 150 μM. The prepared peptide solutions were added to cuvettes with a light path of 1 mm, and the ellipticities of the peptides in a wavelength range from 190 nm to 250 nm were measured using a J-820 (Jasco, Tokyo, Japan) spectropolarimeter at a scan rate of 1 nm/s. The average residue ellipticity was calculated via the following equation:

θ*_M_* = (θ*_obs_* × 1000)/(*c* × *l* × *n*)

where θ*_M_* refers to the average residue ellipticity (deg cm^2^ dmol^−1^), θ*_obs_* is the measured ellipticity (mdeg), *c* is the peptide concentration (mM), *l* is the path length (mm) and *n* is the number of amino acids.

### 4.4. Measurement of Hemolytic Activity

Briefly, collected human red blood cells (hRBCs) were washed three times with PBS (pH 7.4) and diluted to 1% in PBS. Next, equal volumes of blood cells were mixed with different peptide concentrations in 96-well plates followed by incubated at 37 °C for 1 h. The mixtures were centrifuged (1000 g, 5 min), and the supernatants were transferred to 96-well plates to measure the optical density (OD) at 570 nm. Untreated blood cells were used as negative controls, and 0.1% Triton X-100-treated blood cells were used as positive controls. The minimum peptide concentration corresponding to 5% hemolytic activity was defined as the minimal hemolytic concentration (MHC). These assays were independently repeated three times.

### 4.5. Measurement of Cytotoxicity

According to a previously described method, the cytotoxicity of each peptide against the RAW264.7 murine macrophage cell line and human embryonic kidney (HEK) 293T cells was determined by measuring the MTT dye reducibility [47]. Briefly, 1 × 10^4^ cells were cultured in DMEM containing 10% fetal bovine serum and incubated for 4 h at 37 °C under 5% CO_2_. Approximately 50 μL aliquots of cell suspension were mixed with 50 μL aliquots of solutions containing different peptide concentrations in 96-well plates (final concentrations ranging from 0.5–128 μM) followed by further incubation for 4 h under the same conditions. Subsequently, 50 μL of MTT (0.5 mg/mL) was directly added, and incubation was continued for 4 h. The cell cultures were centrifuged (1000 g, 5 min), and the supernatant was discarded. Next, 150 μL of DMSO was added to dissolve the crystals. The absorbance was measured at 570 nm in a microplate reader (Tecan GENios F129004, Tecan, Austria).

### 4.6. Minimum Inhibitory Concentration (MIC) Measurements

The minimum inhibitory concentration (MIC) was measured using a broth microdilution method to determine the in vitro antimicrobial activity of the peptides [5]. Bacteria were inoculated into MHB and incubated overnight at 37 °C until the bacteria were in log phase growth. Next, we corrected the bacterial concentration to approximately 1 × 10^5^ CFU/mL using MHB. The peptides were added to 96-well plates containing 0.01% acetic acid and 0.2% bovine serum albumin at final concentrations ranging from 0.25 to 128 μM. After incubation at 37 °C for 24 h, the absorbance was measured at 492 nm using a microplate reader (Tecan GENios F129004, Tecan, Austria). Broth containing microbes and microbe-free cells were used as positive and negative controls, respectively. The lowest peptide concentration with no increase in absorbance compared to that of the negative control was defined as the MIC. The experiments were independently performed three times.

### 4.7. Salt Sensitivity Assays

The effects of salt ions on the antibacterial activities of the antimicrobial peptides were evaluated. Different salt ion concentrations were added to BSA peptide dilutions, and the next steps followed the MIC measurement method. The final salt ion concentrations were: 150 mM NaCl, 4.5 mM KCl, 6 μM NH_4_Cl, 8 μM ZnCl_2_, 1 mM MgCl_2_ and 4 μM FeCl_3_. These tests were independently repeated three times.

### 4.8. Antimicrobial Mechanism Assays

The mechanism of action of the antimicrobial peptides was further elucidated via scanning electron microscopy (SEM, Bectone-Dickinson, San Jose, CA, USA) to observe changes in the surface of the bacteria after treatment with the antimicrobial peptides. To further investigate the interaction between the peptides and the bacterial membrane, a series of experiments were performed.

#### 4.8.1. LPS Binding Assay

The abilities of the antimicrobial peptides to bind LPS were determined using the fluorescent dye BODIPY-TR-cadaverine (BC) substitution method. LPS (50 μg/mL) from *E. coli* O111:B4 was incubated with BC (5 μg/mL) in Tris buffer (50 mm, pH 7.4) for 4 h. Next, 50 μL of various concentrations of peptides (final concentration of 1–64 μM) and 50 μL of LPS-BC mixture were added to 96-well plates, and the fluorescence was measured using a fluorescence spectrophotometer (Infinite 200 pro, Tecan, China) at an excitation wavelength of 580 nm and an emission wavelength of 620 nm. Each test was performed three times. The values were converted to %∆F (AU) using the following equation:

%ΔF (AU) = [(F*_obs_* − F_0_)/(F_100_ − F_0_)] × 100

where F*_obs_* is the fluorescence value measured in the presence of the peptide, F_0_ is the initial fluorescence of LPS with BC without the addition of peptides, F_100_ is the fluorescence value produced by adding 10 μg/mL polymyxin B.

#### 4.8.2. Outer Membrane Permeability Assay

The NPN uptake assay was used to assess the penetrating ability of the peptides into the outer membrane as previously described [48]. Briefly, *E. coli* ATCC25922 was incubated in MHB to mid-log phase. The cells were washed thrice with HEPES buffer (pH 7.4, containing 5 mM glucose), and the concentration was diluted to 10^5^ CFU/mL. Next, a final concentration of 10 μM NPN was added followed by incubation for 30 min at room temperature in the dark. An equal volume of the bacterial liquid and different peptide concentrations were mixed in black 96-well plates, and the fluorescence intensities of the samples were measured using an F-4500 fluorescence spectrophotometer (Hitachi, Tokyo, Japan) at an excitation wavelength of 350 nm and an emission wavelength of 420 nm. The values were converted to percent NPN uptake using the equation:

% NPN uptake = (F_obs_ − F_0_)/(F_100_ − F_0_) × 100

where the fluorescence value of Fobs measured in the presence of the peptide, F_0_ is the initial fluorescence value of the solution containing NPN, and F_100_ is the fluorescence value produced by adding 10 μg/mL polymyxin B.

#### 4.8.3. Inner Membrane Permeability Assay

Changes in intracellular membrane permeability were assessed by measuring the intracellular β-galactosidase activity as previously described. Briefly, *E. coli* ATCC25922 cells were cultured in MHB containing 2% lactose at 37 °C, harvested by centrifugation, washed three times, and resuspended in 5 mM HEPES endomembrane buffer with 1.5 mM ONPG at a final concentration of 0.05 at OD 600 nm achieved via dilution. The peptides were diluted with 5 mM HEPES (containing 20 mM glucose, pH 7.4) buffer and added to 96-well plates at final concentrations ranging from 0.5 to 64 μM, after which the prepared bacterial solution was added to the desired wells. The OD measurements were measured from 0 to 3480 s at 420 nm, with a measurement every 120 s, and the ONPG influx was used as an indicator of cell permeability.

#### 4.8.4. Membrane Depolarization

The effects of the antimicrobial peptides on the plasma membrane potential were determined used cell membrane potential-sensitive fluorescent dye diSC_3_-5. Briefly, *E. coli* ATCC25922 cells were incubated in MHB to mid-log phase at 37 °C, rinsed 3 times with HEPES buffer, adjusted to an OD600 of 0.05. Next, KCl was used to adjust the final concentration to 0.4 μM and diSC_3_-5 was added. The mixture was incubated for 30 min at 37 °C. The antibacterial peptides at concentrations of 0.5, 1 and 2×MIC were then added to a cuvette containing 2 mL of bacterial solution, and the fluorescence change was recorded from 0 to 600 s with an F-4500 fluorescence spectrophotometer (Hitachi, Tokyo, Japan) (excitation λ = 622 nm, emission λ = 670 nm). Bacteria without antimicrobial peptides were used to determine the background fluorescence.

#### 4.8.5. Swimming Motility Assay

The cell motility assay was performed on LB medium containing 0.3% agar [49,50]. The peptides were added to 10 mL of medium at final concentrations of 1–8 μM. After mixing well, the mixtures were poured into 6-well plates and dried for 2 h. Subsequently, an overnight culture of *E. coli* ATCC 25922 was inoculated in the center of the movement plate. After incubation at 37 °C for 20 h, images were taken using a FluoroChemQ system (Cell biosciences, San Jose, CA, USA). The experiment was independently repeated three times.

#### 4.8.6. SEM Characterization

For the SEM sample preparation, *E. coli* ATCC25922 cells were cultured at 37 °C to mid-log phase in MH broth with constant shaking at 220 rpm, centrifuged at 1000 g and then adjusted to an OD600 of 0.1–0.2 in PBS. Peptides at 1 MIC were added to the bacterial solution followed by incubation at 37 °C for 1 h. Bacteria incubated with PBS served as controls. After the incubation, the mixture was centrifuged at 5000 g for 5 min, and the collected bacteria were washed three times with PBS. Approximately 2.5% (*w*/*v*) glutaraldehyde was added for fixation at 4 °C overnight, followed by washing three times with PBS. The samples were dehydrated with 50%, 70%, 90%, 100% ethanol for 10 min. A mixture of ethanol and tert-butanol was added to the dried bacterial cells for 30 min, and then t-butanol was added for 1 h. Finally, the samples were dehydrated and coated with liquid CO_2_ in a critical point dryer and then observed using a Hitachi S-4800 SEM (Hitachi, Tokyo, Japan).

#### 4.8.7. Inhibition of Endotoxin-Induced Inflammation

TNF-α production by the RAW264.7 cells was stimulated with LPS to evaluate the LPS neutralization characteristics of the peptides [51]. Briefly, (1.0–2.0) × 10^5^ RAW264.7 cells were plated in 96-well plates and stimulated with LPS (100 ng/mL) for 18 h at 37 °C in the absence or presence of peptides (2–64 μM). The negative and positive controls were untreated cells and cells treated with LPS alone. The supernatant was used to evaluate the endotoxin neutralizing ability of the antimicrobial peptides by analyzing the TNF-α production using Griess reagent (Promega, Madison, WI, USA) and ELISA (Boster, Wuhan, China).

### 4.9. Statistical Analysis

Data were analyzed by ANOVA using SPSS 18.0 software, and significant differences between the means were evaluated by Tukey’s test for multiple comparisons. Quantitative data are presented as the mean ± standard error of the mean. *p* < 0.01 was considered to be very statistically significant.

## 5. Conclusions

In this study, a series of β -hairpin peptides derived from PG-1 were designed. The results of repeated β-hairpin of simple amino acids showed that the different subtypes of hydrophobic amino acids had a significant effect on the antibacterial activity and hemolysis of peptides. HV2 had better selectivity for Gram-negative bacteria without hemolysis. Peptides were non-structural in aqueous solution, but they fold into β-hairpin structure when interacting with TFE. Hydrophobic amino acids of different subtypes significantly affected the antibacterial activity of AMP, and the cell selectivity of the peptide HV2 was significantly higher than that of PG-1. HV2 showed a strong permeabilized outer layer, depolarizing the plasma membrane and the ability to permeabilize the inner membrane, revealing that HV2 kills the bacterial cell membrane by destroying the cell membrane in a detergent-like mechanism, resulting in bacterial cell membrane leakage. The action mechanism of peptides on Gram-negative bacteria was analyzed by scanning electron microscope (SEM), which revealed the mechanism of killing bacterial cells by destroying the cell membrane, resulting in the leakage of the cytoplasm, and finally leading to the dissolution of the cells. In addition, HV2 had a strong binding capacity with LPS, thereby reducing the production of inflammatory factors by cells. Therefore, HV2 had the potential as a novel antibacterial agent against Gram-negative bacteria. In this study, we provided a design approach for a new idea involving template peptides and supported the development of this simplified and optimized natural peptides design.

## Figures and Tables

**Table 1:** Peptides design and key physicochemical parameters.

Peptides	Sequence	Theoretical MW^a^	Measured MW	Retention Time (min)	H^b^	μHrel^c^	Net Charge
HI2	RRIHIHI^D^PGIHIHIRR-NH_2_	2023.46	2024.47	11.13	−0.35	2.39	+9
HW2	RRWHWHW^D^PGWHWHWRR-NH_2_	2461.78	2461.82	10.55	0.02	2.35	+9
HV2	RRVHVHV^D^PGVHVHVRR-NH_2_	1939.29	1939.33	12.20	−2.07	1.89	+9
HF2	RRFHFHF^D^PGFHFHFRR-NH_2_	2227.56	2227.59	18.06	0.13	2.53	+9
PG-1	RGGRLCYCRRFCVCVGR-NH_2_	2155.61	2156.35	12.56	−2.42	1.37	+7

^a^ Molecular weight (MW) was measured by mass spectroscopy (MS). ^b^ The mean hydrophobicity (H) is the total hydrophobicity (sum of all residue hydrophobicity indices) divided by the number of residues. ^c^ The relative hydrophobic moment (μHrel) of a peptide is its hydrophobic moment relative to that of a perfectly amphipathic peptide. This gives a better idea of the amphipathicity using different scales.

**Table 2:** MIC, MHC and TI of the peptides.

	HI2	HW2	HV2	HF2	PG-1
MIC^a^ (μM)					
*Gram-*					
*E. coli* 25922	32	4	8	4	4
*E. coli* K88	32	4	4	2	2
*E. coli* K99	>128	4	8	4	2
*E. coli* UB1005	16	2	8	8	2
*S.pullorum* C7913	16	2	8	4	4
*P. auruginosa* 27853	>128	8	8	>128	8
*Gram+*					
*S.aureus* 29213	>128	4	>128	64	2
*S.aureus* 43300	>128	2	>128	4	8
*S.epidermidis* 12228	>128	2	>128	4	8
MBC^b^ (GM)					
*Gram (−)*	50.80	3.56	7.13	8.00	3.17
*Gram (+)*	256	2.52	256	10.08	5.04
*Gram (+,−)*	87.09	3.17	23.52	8.64	3.70
MHC^c^(µM)	>128	8	>128	16	4
TI^d^					
TI(−)	5.04	2.25	35.90	2.00	1.26
TI(+)	1	3.17	1	1.59	0.79
TI(+,−)	2.94	2.52	10.88	1.85	1.08

^a^ Minimum inhibitory concentrations (MIC) were determined as the lowest concentration of peptides that prevented visible turbidity. ^b^ The geometric mean (GM) of the peptides MICs against all four bacterial strains was calculated. When no detectable antimicrobial activity was observed at 128 μM, a value of 256 μM was used to calculate the therapeutic index. ^c^ MHC is the minimum hemolytic concentration that caused 5% hemolysis of human red blood cells (hRBC). When no detectable hemolytic activity was observed at 128 μM, a value of 256 μM was used to calculate the therapeutic index. ^d^ Therapeutic index (TI) is the ratio of the MHC to the geometric mean of MIC (GM). Larger values indicate greater cell selectivity.

**Table 3:** MIC values of peptides in the presence of physiological salts. ^a^

Peptides	Control ^b^	NaCl ^b^	KCl ^b^	NH_4_Cl ^b^	MgCl_2_^b^	ZnCl_2_^b^	FeCl_3_^b^
Gram-negative strain *E. coli* ATCC25922							
HV2	8	4	4	4	8	1	4
PG-1	4	4	4	4	4	2	4

^a^ Minimum inhibitory concentrations (MIC) were determined as the lowest concentration of the peptides that inhibited bacteria growth. ^b^ The final concentrations of NaCl, KCl, NH_4_Cl, MgCl_2_, ZnCl_2_, and FeCl_3_ were 150 mM, 4.5 mM, 6 μM, 1 mM, 8 μM, and 4 μM, respectively, and the control MIC values were determined in the absence of these physiological salts.

## Supplementary Materials

Supplementary materials can be found at https://www.mdpi.com/1422-0067/20/16/3954/s1.
