# Supplementary material for: Bioactivity and Bactericidal Mechanism of Histidine-Rich β-Hairpin Peptide Against Gram-Negative Bacteria
Source: Int J Mol Sci. 2019 Aug 14;20(16):3954. doi: 10.3390/ijms20163954 (PMC6718988; doi:10.3390/ijms20163954)
Supplement: Supplementary file 1 [file ijms-20-03954-s001.pdf]

Supporting information

Title:

Bioactivity and bactericidal mechanism of histidine-rich β-hairpin peptide against Gram-negative bacteria

Authors:

Na Dong, Chensi Wang, Tingting Zhang, Lei Zhang, Chenyu Xue, Xinjun Feng, Chongpeng Bi and Anshan Shan*

Contents of supporting information

Supplementary Figure S1: MALDI-TOF MS of the engineered peptides............................**S2-S3**

Supplementary Figure S2: HPLC spectra of the engineered peptides.................................**S4-S6**

Supplementary Figure S3: The Cytotoxicity of peptides against HEK 293T cells.....................**S7**

Supplementary Table S1: The MICsa (μM) of HV2, Polymyxin B, Gentamicin, Vancomycin and Ceftazidime against Bacteria..**S8**

HI2

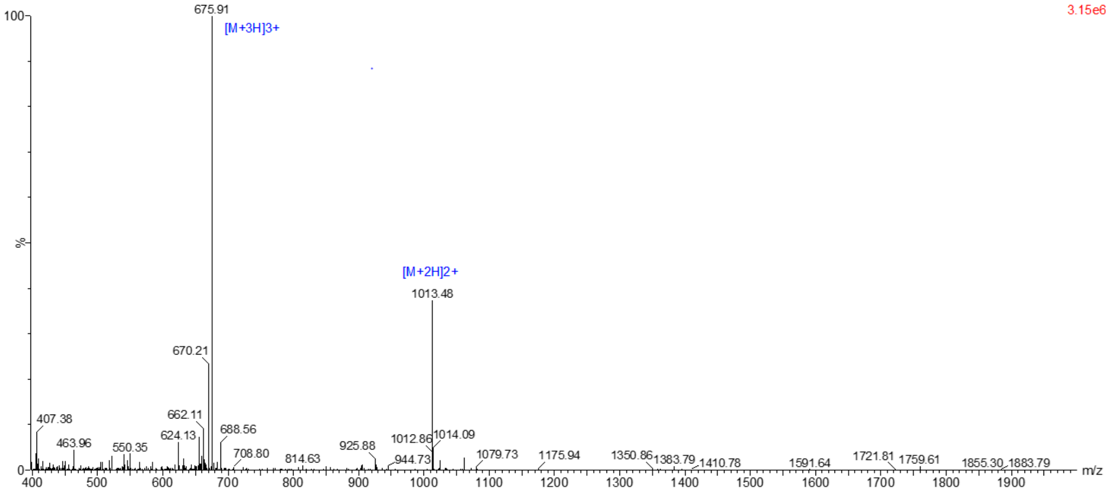

675.91
[M+3H]3+
3.15e6
[M+2H]2+
1013.48
670.21
407.38
662.11
688.56
463.96
624.13
550.35
708.80
814.63
925.88
1012.86
1014.09
944.73
1079.73
1175.94
1350.86
1383.79
1410.78
1591.64
1721.81
1759.61
1855.30
1883.79
%
m/z

HV2

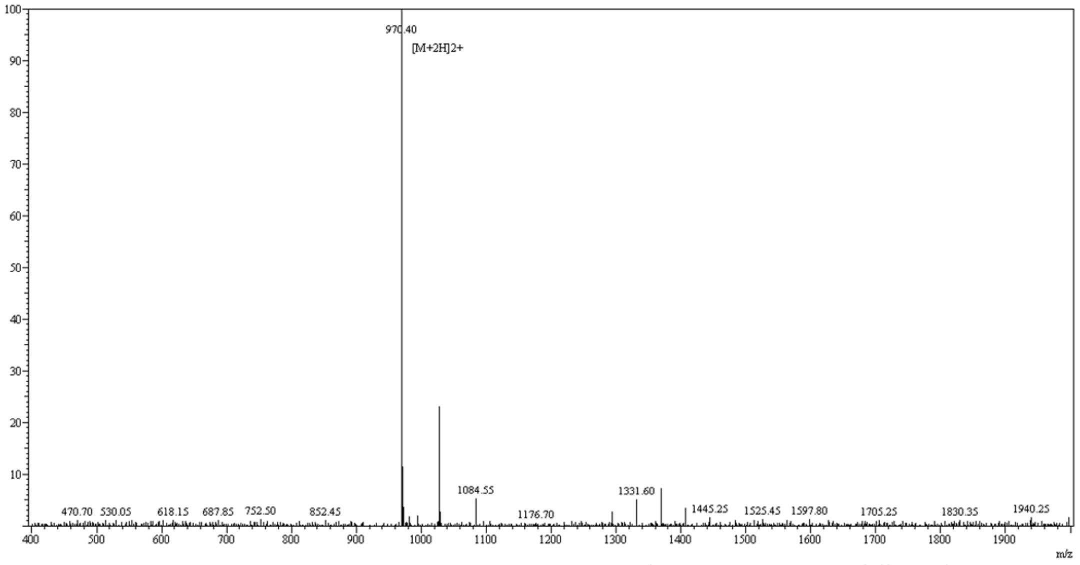

970.40
[M+2H]2+
1084.55
1331.60
470.70
530.05
618.15
687.85
752.50
852.45
1176.70
1445.25
1525.45
1597.80
1705.25
1830.35
1940.25
m/z

HW2

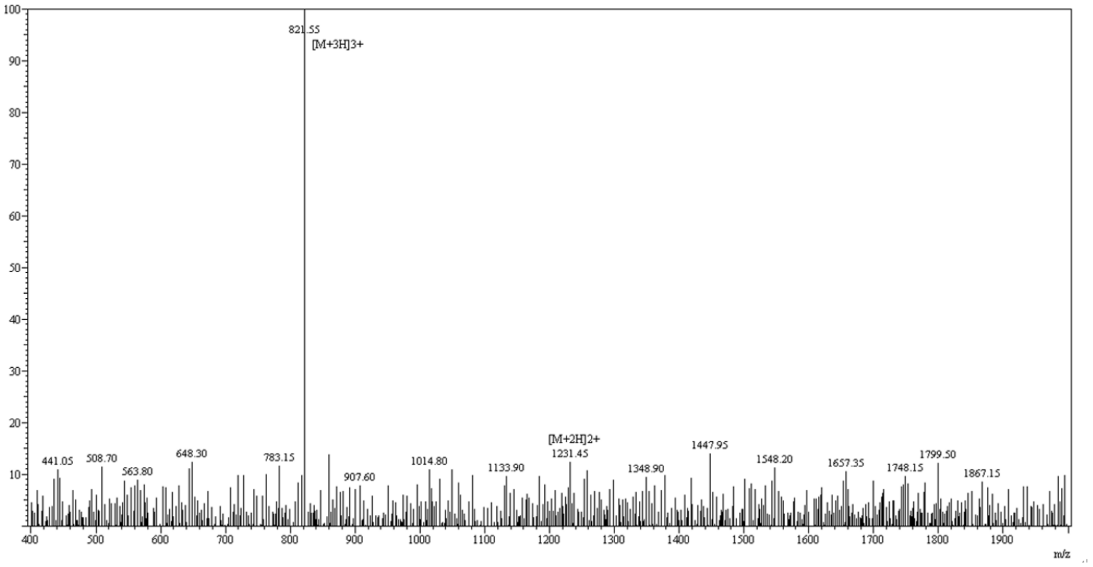
821.55
[M+3H]3+
[M+2H]2+
1231.45
441.05
508.70
563.80
648.30
783.15
907.60
1014.80
1133.90
1348.90
1447.95
1548.20
1657.35
1748.15
1799.50
1867.15
m/z

HF2

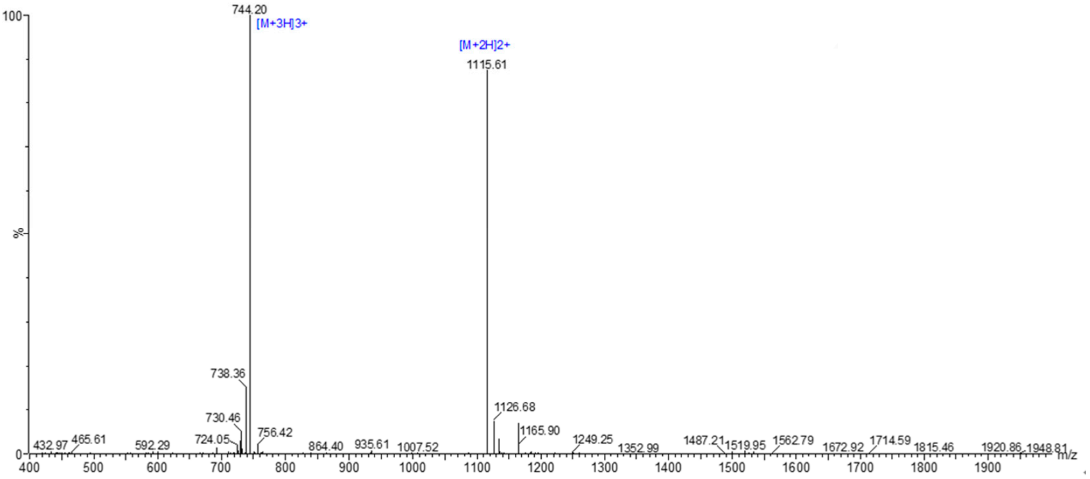
744.20
[M+3H]3+
[M+2H]2+
1115.61
%
738.36
730.46
724.05
756.42
1126.68
1165.90
432.97
465.61
592.29
864.40
935.61
1007.52
1249.25
1352.99
1487.21
1519.95
1562.79
1672.92
1714.59
1815.46
1920.86
1948.81
m/z

PG-1

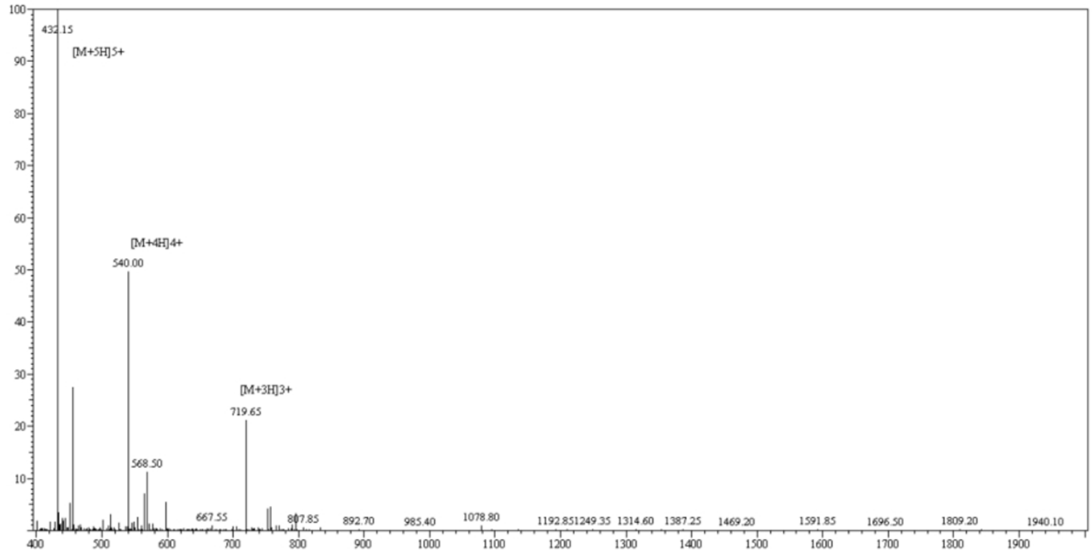

Supplementary Figure S1: MALDI-TOF MS of the engineered peptides

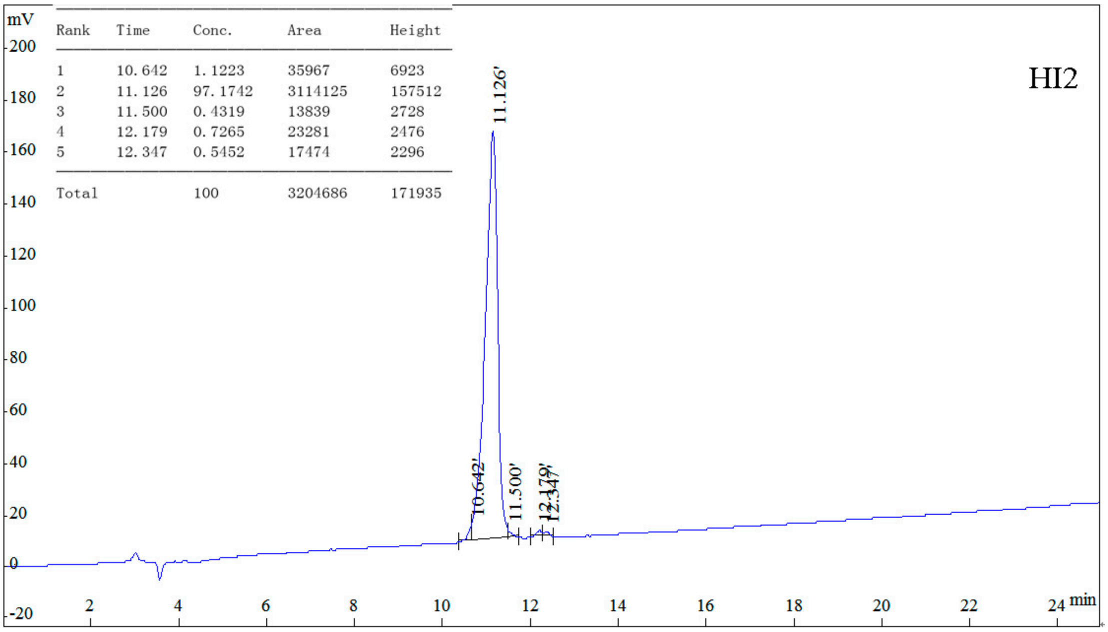

Rank	Time	Conc.	Area	Height
1	10.642	1.1223	35967	6923
2	11.126	97.1742	3114125	157512
3	11.500	0.4319	13839	2728
4	12.179	0.7265	23281	2476
5	12.347	0.5452	17474	2296
Total		100	3204686	171935

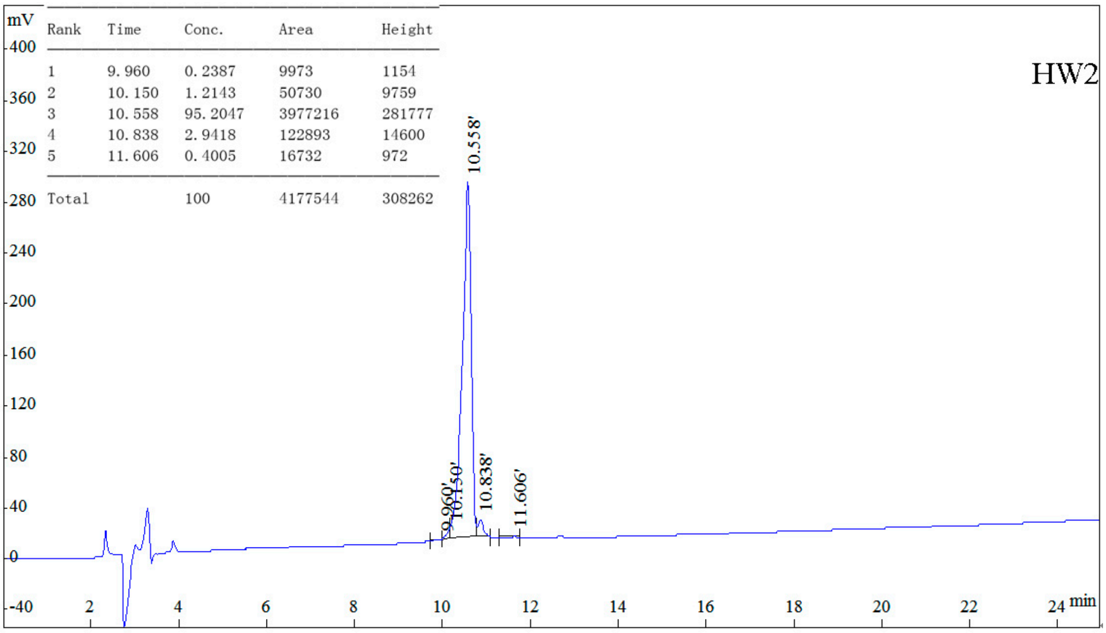

Rank	Time	Conc.	Area	Height
1	9.960	0.2387	9973	1154
2	10.150	1.2143	50730	9759
3	10.558	95.2047	3977216	281777
4	10.838	2.9418	122893	14600
5	11.606	0.4005	16732	972
Total		100	4177544	308262

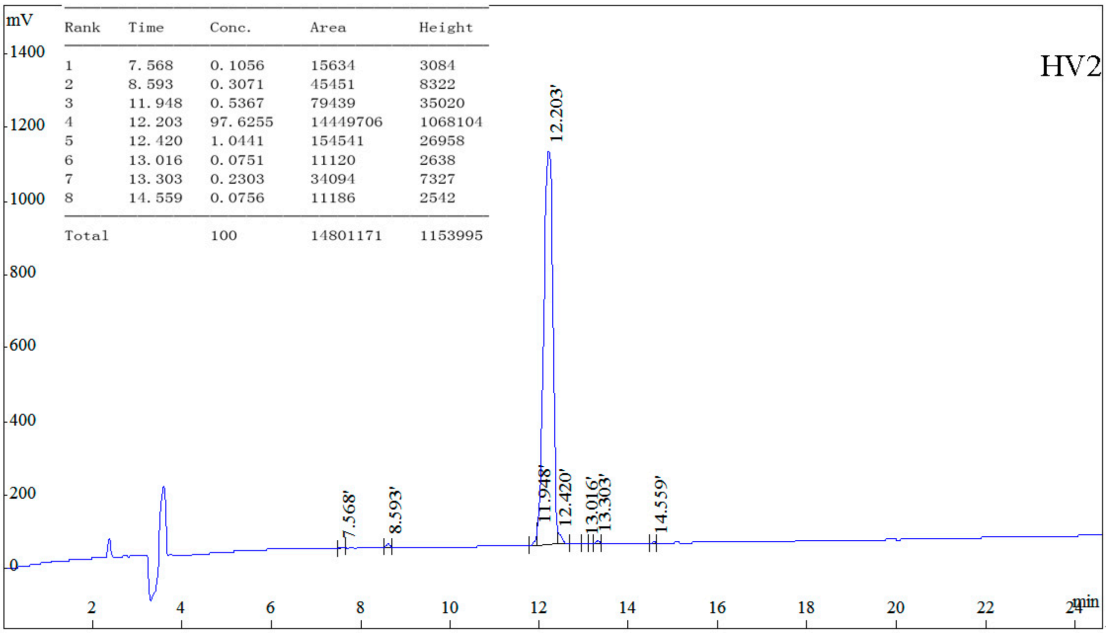

Rank	Time	Conc.	Area	Height
1	7.568	0.1056	15634	3084
2	8.593	0.3071	45451	8322
3	11.948	0.5367	79439	35020
4	12.203	97.6255	14449706	1068104
5	12.420	1.0441	154541	26958
6	13.016	0.0751	11120	2638
7	13.303	0.2303	34094	7327
8	14.559	0.0756	11186	2542
Total		100	14801171	1153995

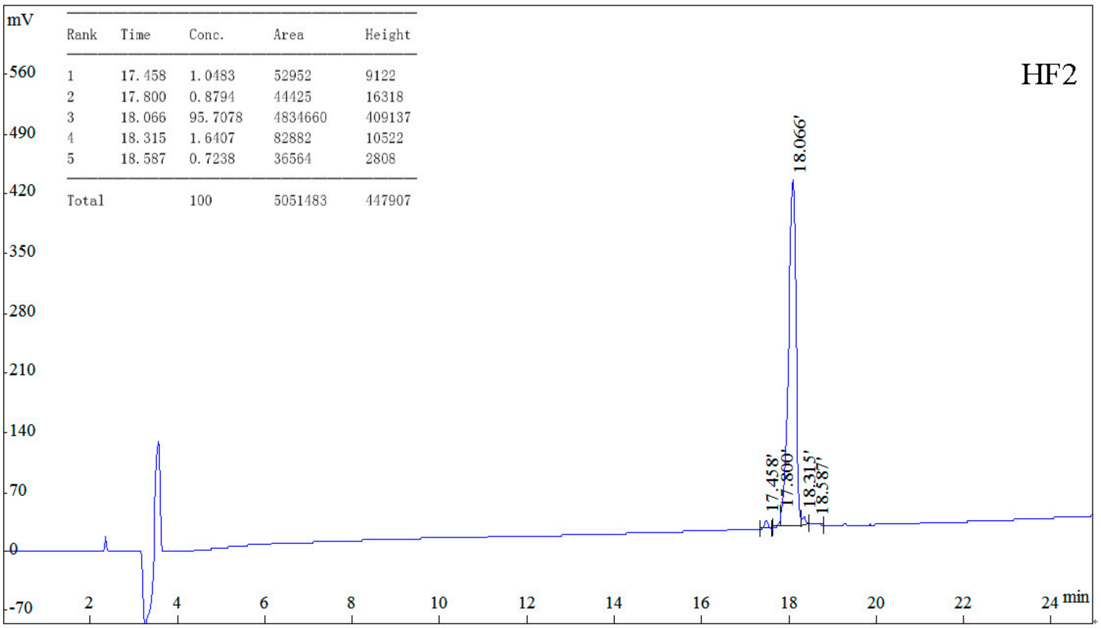

Rank	Time	Conc.	Area	Height
1	17.458	1.0483	52952	9122
2	17.800	0.8794	44425	16318
3	18.066	95.7078	4834660	409137
4	18.315	1.6407	82882	10522
5	18.587	0.7238	36564	2808
Total		100	5051483	447907

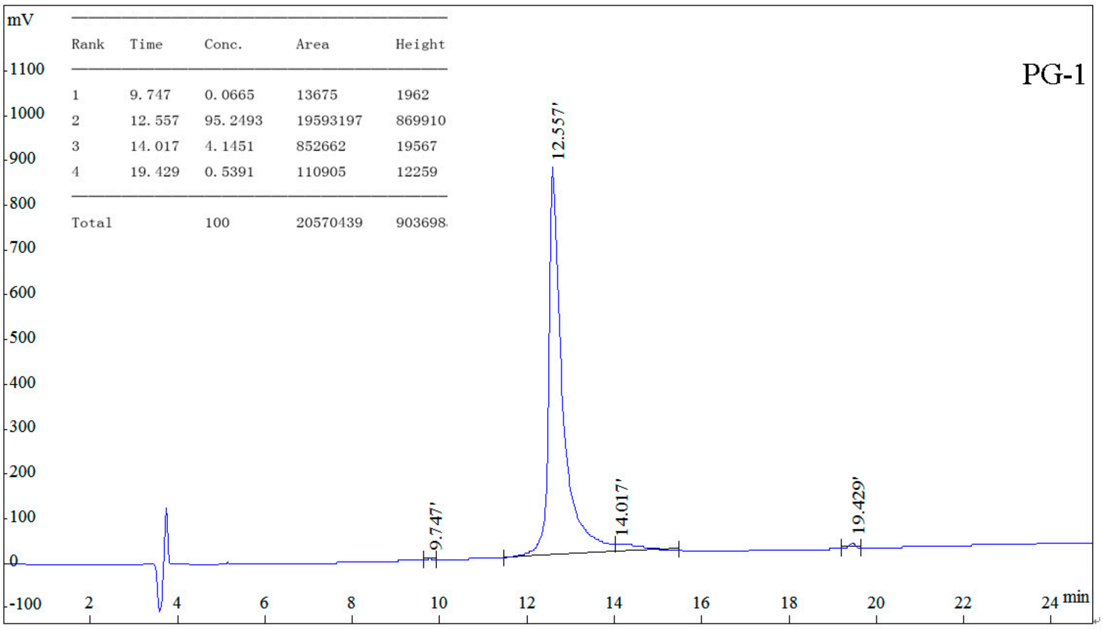

Supplementary Figure S2: HPLC spectra of the engineered peptides

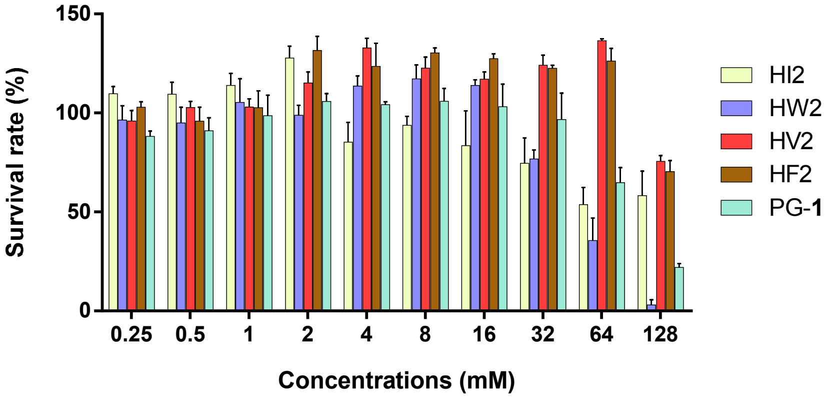

Supplementar Figure S3: Cytotoxicity of peptides against HEK 293T cells

Supplementary Table S1: The MICs[a] of HV2, Polymyxin B, Gentamicin, Vancomycin and Ceftazidime against Bacteria

	HV2	Polymyxin B	Gentamicin	Vancomycin	Ceftazidime
MIC(μM)					
Gram-					
E.coli 25922	8	2	1	8	2
E. coli K88	4	0.5	1	2	1
E. coli K99	8	1	0.5	4	0.5
E.coli UB1005	8	1	0.5	16	1
S.pullorum C7913	8	1	1	8	1
P. auruginosa 27853	8	2	1	>128	2
Gram+					
S.aureus 29213	>128	64	1	0.5	16
S.aureus 43300	>128	64	8	0.5	16
S.epidermidis 12228	>128	32	1	0.5	16
MBC[b]（GM）					
Gram (-)	7.13	1.12	0.79	11.31	1.12
Gram (+)	256	50.8	2	0.5	16
Gram (+,−)	23.52	4	1.08	4	2.72

[a] Minimum inhibitory concentrations (MIC) were determined as the lowest concentration of peptides that prevented visible turbidity.
[b] The geometric mean (GM) of the peptides MICs against all four bacterial strains was calculated. When no detectable antimicrobial activity was observed at 128 μM, a value of 256 μM was used to calculate the therapeutic index.
